# Probing the Long- and Short-Range Structural Chemistry in the C-Type Bixbyite Oxides Th_0.40_Nd_0.48_Ce_0.12_O_1.76_, Th_0.47_Nd_0.43_Ce_0.10_O_1.785_, and Th_0.45_Nd_0.37_Ce_0.18_O_1.815_ via Synchrotron X-ray Diffraction and Absorption Spectroscopy

**DOI:** 10.1021/acsomega.4c02200

**Published:** 2024-06-12

**Authors:** Gabriel L. Murphy, Elena Bazarkina, Volodymyr Svitlyk, André Rossberg, Shannon Potts, Christoph Hennig, Maximilian Henkes, Kristina O. Kvashnina, Nina Huittinen

**Affiliations:** † Institute of Energy and Climate Research (IEK-6), Forschungszentrum Jülich GmbH, Jülich 52428, Germany; ‡ Institute of Resource Ecology, Helmholtz Zentrum Dresden Rossendorf, Dresden 01328, Germany; § The Rossendorf Beamline at ESRF, The European Synchrotron, CS40220, Grenoble Cedex 9 38043, France; ∥ Institut Laue-Langevin, F-38042, Grenoble Cedex 9 38042, France; ⊥ Institute of Chemistry and Biochemistry, Freie Universität Berlin, Berlin 14195, Germany

## Abstract

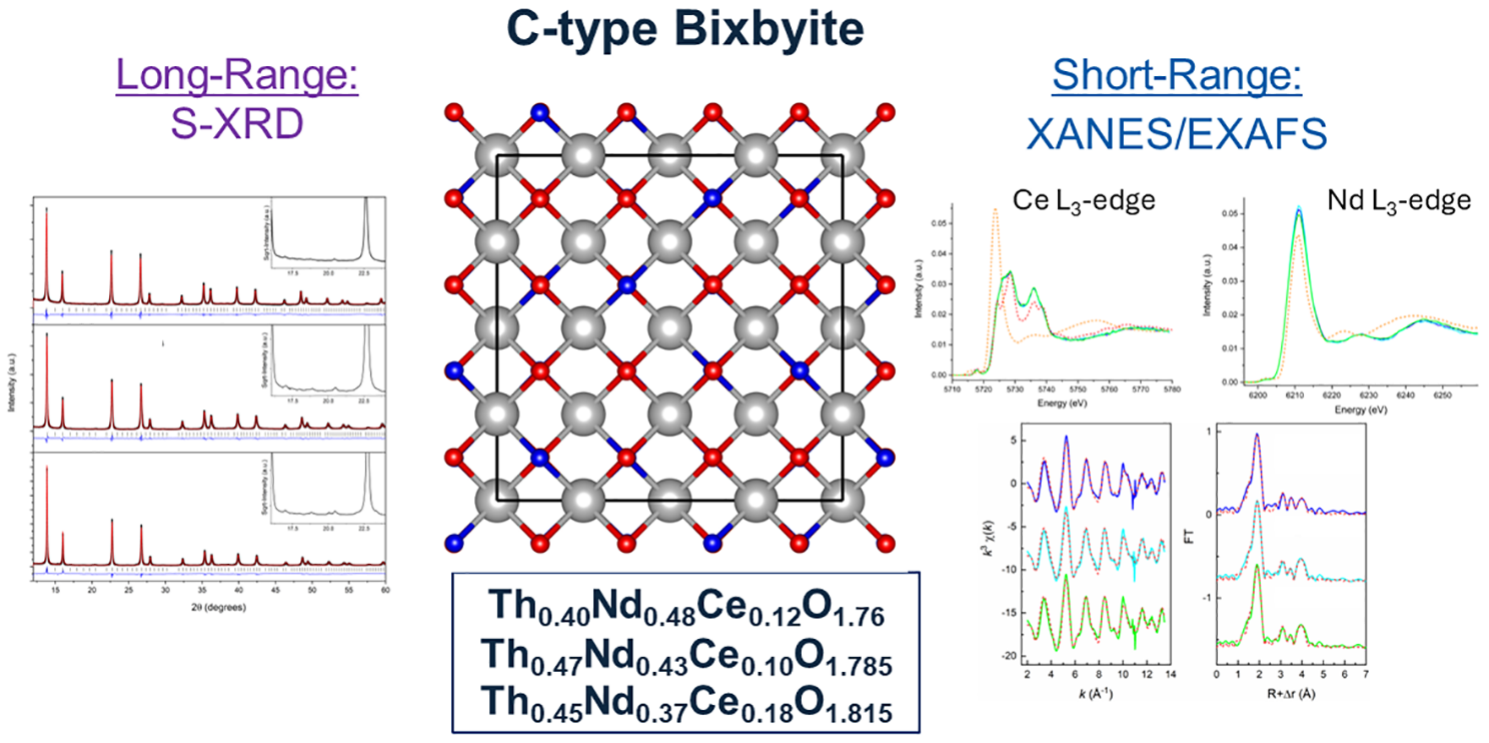

The long- and short-range structural chemistry of the C-type bixbyite compounds Th_0.40_Nd_0.48_Ce_0.12_O_1.76_, Th_0.47_Nd_0.43_Ce_0.10_O_1.785_, and Th_0.45_Nd_0.37_Ce_0.18_O_1.815_ is systematically examined using synchrotron X-ray powder diffraction (S-PXRD), high-energy resolution fluorescence detection X-ray absorption near edge (HERFD-XANES), and extended X-ray absorption fine structure spectroscopy (EXAFS) measurements supported by electronic structure calculations. S-PXRD measurements revealed that the title compounds all form classical C-type bixbyite structures in space group *Ia*3̅ that have disordered cationic crystallographic sites with further observation of characteristic superlattice reflections corresponding to oxygen vacancies. Despite the occurrence of oxygen vacancies, HERFD-XANES measurements on the Ce L_3_-edge revealed that Ce incorporates as Ce^4+^ into the structures but involves local distortion that resembles cluster behavior and loss of nearest-neighbors. In comparison, HERFD-XANES measurements on the Nd L_3_-edge supported by electronic structure calculations reveal that Nd^3+^ adopts a local coordination environment similar to the long-range C-type structure while providing charge balancing for the formation of oxygen defects. Th L_3_-edge EXAFS analysis reveals shorter average Th–O distances in the title compounds in comparison to pristine ThO_2_ in addition to shorter Th–O and Th–Ce distances compared to Th–Th or Ce–Ce in the corresponding F-type binary oxides (ThO_2_ and CeO_2_). These distances are further found to decrease with the increased Nd content of the structures despite simultaneous observation of the overall lattice structure progressively expanding. Linear combination calculations of the M-O bond lengths are used to help explain these observations, where the role of oxygen defects, via Nd^3+^ incorporation, induces local bond contraction and enhanced Th cation valence, leading to the observed increased lattice expansion with progressive Nd^3+^ incorporation. Overall, the investigation points to the significance of dissimilar cations exhibiting variable short-range chemical behavior and how it can affect the long-range structural chemistry of complex oxides.

## 1 Introduction

Oxygen-deficient fluorite compounds and their derivatives have been the focus of a plethora of solid-state materials science investigations across a wide variety of disciplines owing to the tremendous properties they can exhibit stemming from their subtle structural chemistries.^1−3^ This is exemplified in applications pertaining to batteries,^4^ oxide ion conductors,^5^ and ferroelectrics,^6^ among others. Supporting the development of these materials, fundamental investigations on the solid-state chemistry of these compounds, particularly chemical changes including mechanisms that may lead to phase transformations or structural aberrations, have drawn protracted attention from material science communities.^1,3,6−11^ Core to this, fundamental chemical research is exploring and uncovering the unique individual roles and effects constituent elements of these compounds have upon chemical behavior at both the short and long ranges. Such bottom–up approaches have been successful in related materials including pyrochlore,^12,13^ among other complex oxides,^14^ and promote their application toward more exotic material types, such as those stemming from nuclear energy and waste compounds.

**Figure 1:**
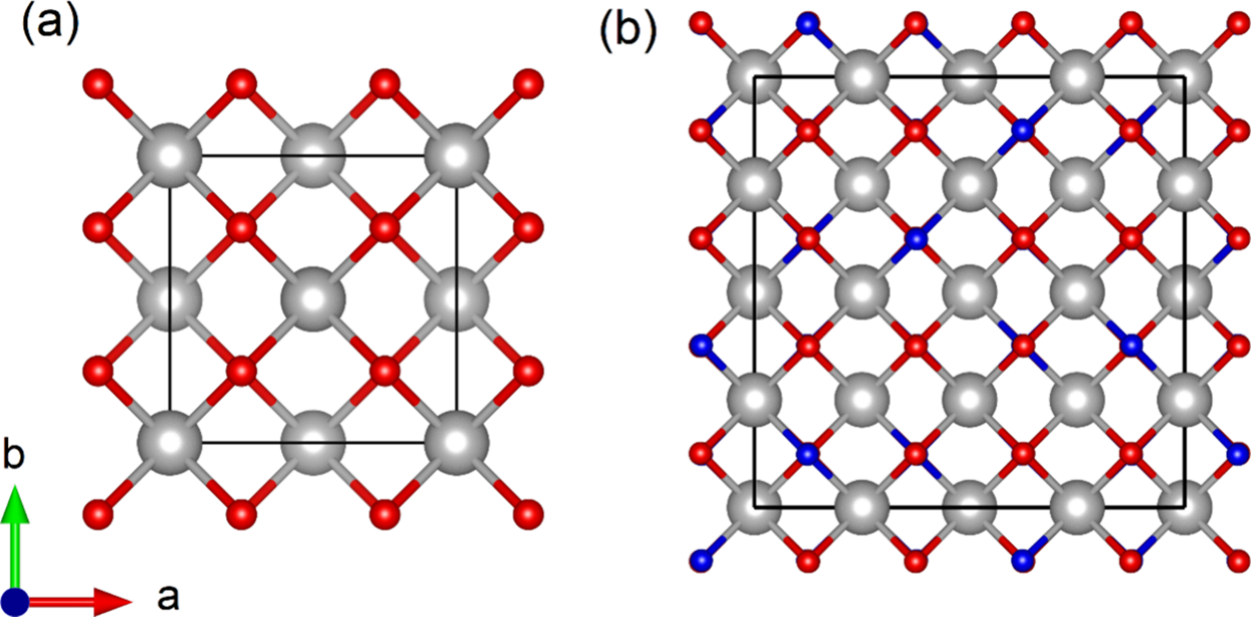
Comparison of (a) F-type fluorite (space group *Fm*3̅*m*) and (b) C-type bixbyite (space group *Ia*3̅) for mixed cation compounds. Uranium and oxygen (O(1)/O(2)) atoms are represented by silver and red/blue spheres, respectively. Note that oxygen vacancy defects are not illustrated in (b).

In the case of fluorite UO_2_ in space group *Fm*3̅*m*, it is the most prevalent fuel source for nuclear power generation globally and is one of the core focuses of spent nuclear fuel (SNF) disposal. After prolonged fuel burnup, the structure has undergone significant changes to its microstructure and crystal lattice due to the occurrence of fission products and radiation damage effects. These processes have been the focus of a plethora of studies regarding the stability of fuels within power reactors and post irradiation when discharged as SNF. Modern fuels have expanded beyond the traditional UO_2_ binary oxide to include oxide variants such as mixed oxide (MOX) fuels, which involve the addition of Pu(IV) into UO_2_, and even ThO_2_ as both standalone or with Pu(IV) addition (TOX) a part of potential fast breeder reactors.^1,15^ Structural-chemically, these materials as fresh fuels are all isostructural as fluorite in the space group *Fm*3̅*m* (F-type). It has been recently shown in Ce-doped UO_2_, U_1–*y*_Ce_*y*_O_2–*x*_, where Ce acts as a surrogate for Pu, that the fluorite structure can undergo phase separation to a C-type bixbyite (space group *Ia*3̅) structure, U_0.54_Ce_0.46_O_2−*x*_ given certain ratios of Ce, U, and O.^16^ Indeed, the separation is not unique to U_1–*x*_Ce_*x*_O_2−*x*_ and has been also observed in U_1–*x*_Gd_*x*_O_2−0.5*x*_ and Th_1–*x*_Gd_*x*_O_2−*x*0.5_.^17^ The transformation to the C-type bixbyite structure from the F-type fluorite in Ce-doped UO_2_ involves the ordering of oxygen vacancy defects, resulting in a doubling of the fluorite lattice parameter, as illustrated comparatively in Figure 1.

Crystallographically, with the doubling of unit cell parameter, the ideal C-type bixbyite structure in space group *Ia*3̅ cations occupies 8b and 24d Wyckoff sites and anions 16c and 48e, whereas the F-type fluorite cations are found on the 4a and anions 8c. The long-range ordering of oxygen vacancies observed in the C-type bixbyite structure typically involves the anionic sites becoming half occupied, whereas the cationic sites are randomly mixed occupied with an infinite correlation length. In the transition from the F-type to the C-type previously identified in U_1–*y*_Ce_*y*_O_2−*x*_,^16^ Ce redox activity is thought to play a central role in stabilizing the structure, where the occurrence of its tetravalent and trivalent oxidation states is suspected of facilitating charge balancing with the ordered oxygen vacancies. Although the cationic lattice in the general C-type bixbyite structure is randomly occupied with mixed cations long-range, local ordering can have a significant effect in charge balancing specific crystallographic sites. In the case of U_1–*y*_Ce_*y*_O_2–*x*_, the chemistry is further complicated by the potential redox activity of U and Ce, where redox behavior resulting in reduction from of oxygen vacancies^18−20^ or oxidation when encountering trivalent cations such as lanthanides in case of U can readily occur.^21^ Conversely for ThO_2_, the chemical situation is somewhat simpler where only Th^4+^ is expected, and in relevant C-type bixbyite oxide derivatives, the redox chemistry is largely driven by the dopant species, such as Ce, allowing the dopant chemistry to be more easily examined.

Several investigations have been performed to explore the solid-state chemistry of ternary Ln^3+^-doped ThO_2_ (Ln^3+^ = trivalent lanthanide cation systems), (Th_1–*x*_Ln_*x*_)O_2–*x*_, compounds.^17,22−24^ Typically, the gradual incorporation of trivalent lanthanides into ThO_2_ results in gradual transformation from the F-type cubic fluorite (space group *Fm*3̅*m*) to the end-member A-type trigonal Ln_2_O_3_ sesquioxide’s (space group *P*3̅*m*1) for Ln = La–Nd.^25,26^ In the case of Sm–Gd, intermediate solid solutions to eventual end member of C-type cubic bixbyite (space group *Ia*3̅) or also B-type monoclinic (space group *C*2/*m*) have been observed,^17,26^ whereas in the case of Tb–Lu and also Y and Sc, C-type bixbyite is found progressively as the end member.^26,27^ In all cases of the C-type bixbyite in the binary sesquioxides, the structures are stoichiometric with respect to oxygen content under oxidizing conditions. In the case of Nd, there are no reports of a C-type bixbyite forming in a solid solution with ThO_2_ for the ternary system. For Ce^4+^ incorporation into ThO_2_, a continuous solid solution behavior is observed for all values of *x,* (Th_1–*x*_Ce_*x*_)O_2_.^28^ The quaternary system (Th_1–*x–y*_Ln^4+^_*x*_Ln^3+^_*y*_)O_2−*y*0.5_ has received less attention, despite acting as a potential surrogate model for SNF MOX/TOX or minor actinide transmutation fuels ((U_1–*x–y*_Pu_*x*_Am_*y*_)O_2−*y*0.5_ | (Th_1–*x−y*_Nd_*x*_Ce_*y*_)O_2−*x*0.5_)^29^ or as a potential oxygen ion conductor. A recent study by Nandi et al.^30^ used laboratory X-ray diffraction and Raman spectroscopy, to examine (Th_1–*x–y*_Nd_*x*_Ce_*y*_)O_2−*x*0.5_ and explore phase separation. They found that a C-type bixbyite structure could be obtained under certain ratios of Th, Nd, and Ce even though previously, no C-type bixbyite was found to occur in either of the individual ternary thorium oxide systems with Ce and Nd, Th_1–*x*_Nd_*x*_O_2−*x*0.5_, Th_1–*x*_Ce_*x*_O_2_, and Ce_1–*x*_Nd_*x*_O_2−*x*0.5_, forming A and F types.^30^ In their study, the C-type bixbyite structure was determined based on the observation of characteristic superlattice reflections from XRD diffractograms, although no formal structural refinement analysis was performed nor was redox determination using relevant spectroscopic techniques.

Considering the relevance of the quaternary (Th_1–*x*–*y*_Nd_*x*_Ce_*y*_)O_2–*x*0.5_ system in exploring the structural behavior and chemistry of SNF-related materials,^1,10,31^ among the broader field of fluorite and actinide structure solid-state chemistry,^32^ it is salient to understand the structural and redox chemistry of C-type bixbyite phases that can form in this family of oxides. Accordingly, the present investigation has examined the C-type bixbyite oxides Th_0.40_Nd_0.48_Ce_0.12_O_1.76_, Th_0.47_Nd_0.43_Ce_0.10_O_1.785_, and Th_0.45_Nd_0.37_Ce_0.18_O_1.815_ via high-resolution short- and long-range techniques including synchrotron X-ray powder diffraction, high-energy resolution fluorescence detection X-ray near edge structure and extended X-ray absorption fine structure spectroscopy measurements. These measurements were used to determine the roles of the Th, Nd, and Ce cations in these C-type bixbyite structures and the effect they have upon the redox chemistry in addition to the long- and short-structural chemistry.

## 2 Experimental Section

### 2.1 Synthesis

Th_0.40_Nd_0.48_Ce_0.12_O_1.76_, Th_0.47_Nd_0.43_Ce_0.10_O_1.785_, and Th_0.45_Nd_0.37_Ce_0.18_O_1.815_ were synthesized using a coprecipitation method followed by high-temperature solid-state treatment. The compositions were chosen based on a previously proposed phase diagram for the ternary Th–Nd–Ce oxide system.^30^ Using targeted stoichiometric ratios, Th(NO_3_)_4_·5H_2_O, Ce(NO_3_)_4_·6H_2_O and Nd(NO_3_)_3_·6H_2_O aqueous solutions were prepared. To these, 16.5 M ammonia solution was carefully added while stirring, resulting in the precipitation of white solids. A 300% excess of ammonia was used to ensure the completion of the reaction and precipitation.^31^ The precipitates were separated and washed three times with deionized water. After the third wash, water was replaced with ethanol, and the mixture was allowed to dry. The solid precipitates were then calcined at 1000 °C for 24 h using a box furnace and after, collected for later measurement and analysis.

### 2.2 Synchrotron X-ray Powder Diffraction

Ambient temperature synchrotron X-ray powder diffraction (S-PXRD) experiments were performed at the BM20 Rossendorf beamline^33^ (ROBL) at the European Synchrotron Radiation Facility (ESRF), Grenoble, France. Diffraction data were collected on a high-resolution XRD1 machine equipped with a Dectris Pilatus 100,000 photon counting detector. Synthesized samples were finely ground and packed in glass capillaries of 0.3 mm diameter enclosed in 1 mm Kapton tubes that served as confinement barriers. The energy of synchrotron radiation was set at 16,000 eV, just below the Th L_3_ absorption edge of 16 300 eV, and the geometry of the experimental setup was determined using a NIST LaB_6_ standard reference. Experiments were performed in a transmission mode, and corresponding 2D data were reduced using the PyFAI library adapted for diffractometers mounted on a goniometer arm.^34^ Structural analysis was performed using the Rietveld and Le Bail methods as implemented in the program GSAS-II.^35^ The peak shapes were modeled using a pseudo-Voigt function, and the background was estimated using a 6–12 term shifted Chebyschev function. The scale factor, detector zero-point, and lattice parameters were refined together with the peak profile parameters.

### 2.3 High-Energy Resolution Fluorescence Detection X-ray Absorption Spectroscopy

Nd and Ce L_3_-edge HERFD-XANES measurements were performed on Th_0.40_Nd_0.48_Ce_0.12_O_1.76_, Th_0.47_Nd_0.43_Ce_0.10_O_1.785_, and Th_0.45_Nd_0.37_Ce_0.18_O_1.815_ at the ROBL^33^ beamline of the ESRF, BM20, in Grenoble, France. Samples were undiluted and mounted in Kapton sample holders for containment. The incident energy was scanned by using a Si(111) monochromator. HERFD-XANES spectra were collected at room temperature using an X-ray emission spectrometer equipped with five crystal analysers, i.e., Ge(331) for Ce L_3_-edge and Si(333) for Nd L_3_-edge. The X-ray emission spectrometer operated crystal analysers of 1 m bending radius and a silicon drift X-ray detector in a vertical Rowland geometry.^36^ The spectrometer was tuned to maximum X-ray emission line energies of Lα_1_ (4839.2 eV) for Ce and Lβ_2,15_ (6087.5 eV) for Nd. The corresponding Bragg angles were 80.7° for Ce and 77.0° for Nd. The detected intensity was normalized to the incident flux. Beam size was estimated to be 30 μm (vertically) by 2 mm (horizontally). Data analysis was performed by using the software package ATHENA.^37^

### 2.4 Electronic Structure Calculations

The real-space Green’s function code FEFF has been used to calculate the X-ray absorption near edge structure (XANES) in HERFD mode at the Nd L_3_-edge. The XANES-HERFD spectra and the projected density of states were calculated with the ab initio FEFF9.6 code,^38^ based on the full multiple scattering theory. Scattering potentials are calculated by overlapping the free atom densities in the muffin tin approximation and then including the Hedin–Lundqvist self-energy for the excited states. The Hedin–Lundqvist self-energy is used by default in the EXCHANGE card (ixc = 0). The input file (feff.inp) has been constructed by the ATOMS program in Artemis software,^37^ based on the structure of Nd_2_O_3_ (ICSD 645658). The atomic potential is calculated self-consistently using a cluster of radius 8.0 Å. Nd in the bixbyite structure has been made by replacing the Th absorbing atom with Nd (a structure identical to the one used for the EXAFS analysis). The total number of 143 atoms (for bixbyite) and 137 atoms (for Nd_2_O_3_) in a cluster has been used in FEFF calculations.

### 2.5 Extended X-ray Absorption Fine Structure Spectroscopy

EXAFS measurement and analysis were conducted on Th_0.40_Nd_0.48_Ce_0.12_O_1.76_, Th_0.47_Nd_0.43_Ce_0.10_O_1.785_, and Th_0.45_Nd_0.37_Ce_0.18_O_1.815_ at the Th L_3_-edge at the ROBL^33^ beamline of the ESRF, BM20, in Grenoble, France. Measurements were performed at room temperature with an Si(111) monochromator in fluorescence and transmission mode using a Ge-18 elements detector and Ar-filled ionization chambers, respectively, at ambient pressure. The energy calibration was conducted by using the zero crossing of the second derivative transmission signal of the samples at the Th L_3_-edge (assigned as 16,300 eV). Accordingly, the transmission signal was used for the measurement of the samples. Samples were prepared undiluted in Kapton holders for measurement. A minimum of three spectra were collected for each sample. Data treatment including dead time correction, energy calibration, data reduction, averaging of individual scans, and shell fits over a constant *k*-range (2.0–13.5 Å) were conducted with EXAFSPAK.^39^ Theoretical scattering phases and amplitudes were calculated using the ab initio code FEFF8.20.^40^ A Th, Nd, and Ce-substituted bixbyite structure was used as a theoretical model for the fitting of the EXAFS data. During fitting, S_0_^2^ was fixed to 0.9. The coordination numbers of the Th–M (M = Th, Nd, Ce) shells were fixed so that the individual shells reflected the approximate cation stoichiometry in the samples, and the overall Th–M coordination number amounted to 12.

## 3 Results and Discussion

### 3.1 Synchrotron X-ray Powder Diffraction Studies

**Figure 2:**
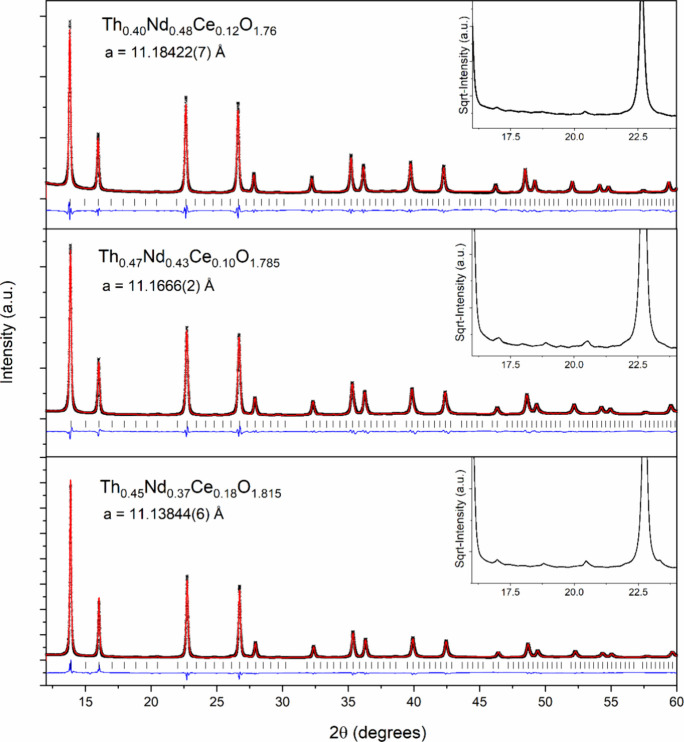
Refinement profiles for Th_0.40_Nd_0.48_Ce_0.12_O_1.76_, Th_0.47_Nd_0.43_Ce_0.10_O_1.785_, and Th_0.45_Nd_0.37_Ce_0.18_O_1.815_ determined from Le Bail refinements against S-PXRD measurements (λ = 0.77563 Å). The insets highlight the characteristic superlattice reflections of the C-type bixbyite structure where the square root of the intensity has been taken to increase clarity. For Th_0.40_Nd_0.48_Ce_0.12_O_1.76_*a* = 11.18422(7) Å, w*R*_p_ = 7.09%, and *R* = 5.35%; Th_0.47_Nd_0.43_Ce_0.10_O_1.785_*a* = 11.1666(2) Å, w*R*_p_ = 5.54%, and *R* = 4.25%; and Th_0.45_Nd_0.37_Ce_0.18_O_1.815_*a* = 11.13844(6) Å, w*R*_p_ = 6.81%, and *R* = 5.49%. Note aberrations in the background were caused by the merging of data sets during post measurement data reduction.

Synthesized samples of Th_0.40_Nd_0.48_Ce_0.12_O_1.76_, Th_0.47_Nd_0.43_Ce_0.10_O_1.785_, and Th_0.45_Nd_0.37_Ce_0.18_O_1.815_ were measured using S-PXRD at room temperature. Careful inspection of diffractograms revealed the occurrence of superlattice reflections consistent with the occurrence of ordered oxygen defect vacancies concordant with the literature for C-type cubic bixbyite.^16^ Nevertheless, the diffraction patterns were analyzed using the Le Bail method where space groups including F-type *Fm*3̅*m*, A-type *P*3̅*m*1, and C-type *Ia*3̅ were trialed. Of these, the only one that returned a satisfactory fit, particularly accounting for the superlattice reflections, was space group *Ia*3̅ corresponding to the C-type bixbyite structure. Figure 2 provides the refinement profiles and insets highlighting the described superlattice reflections for each of the studied compositions. The assignment of Th_0.40_Nd_0.48_Ce_0.12_O_1.76_, Th_0.47_Nd_0.43_Ce_0.10_O_1.785_, and Th_0.45_Nd_0.37_Ce_0.18_O_1.815_ to the C-type bixbyite structure is consistent with the previous work of Nandi et al.,^30^ who examined the (Th_1–*x–y*_Nd_*x*_Ce_*y*_)O_2_–_*y*0.5_ system broadly, providing a relative phase diagram, which the title compounds are consistent with. Interestingly, when the constituents are considered as ternary oxides (Th_1–*x*_Ce_*x*_O_2_, Th_1–*x*_Nd_*x*_O_2–0.5*x*_, Ce_1–*x*_Nd_*x*_O_2–0.5*x*_), C-type bixbyite structures are not reported to occur. This suggests that a degree of entropic mixing is required to assist in structure formation and stabilization.

### 3.2 High-Energy Resolution Fluorescence Detection X-ray Absorption Spectroscopy

**Figure 3:**
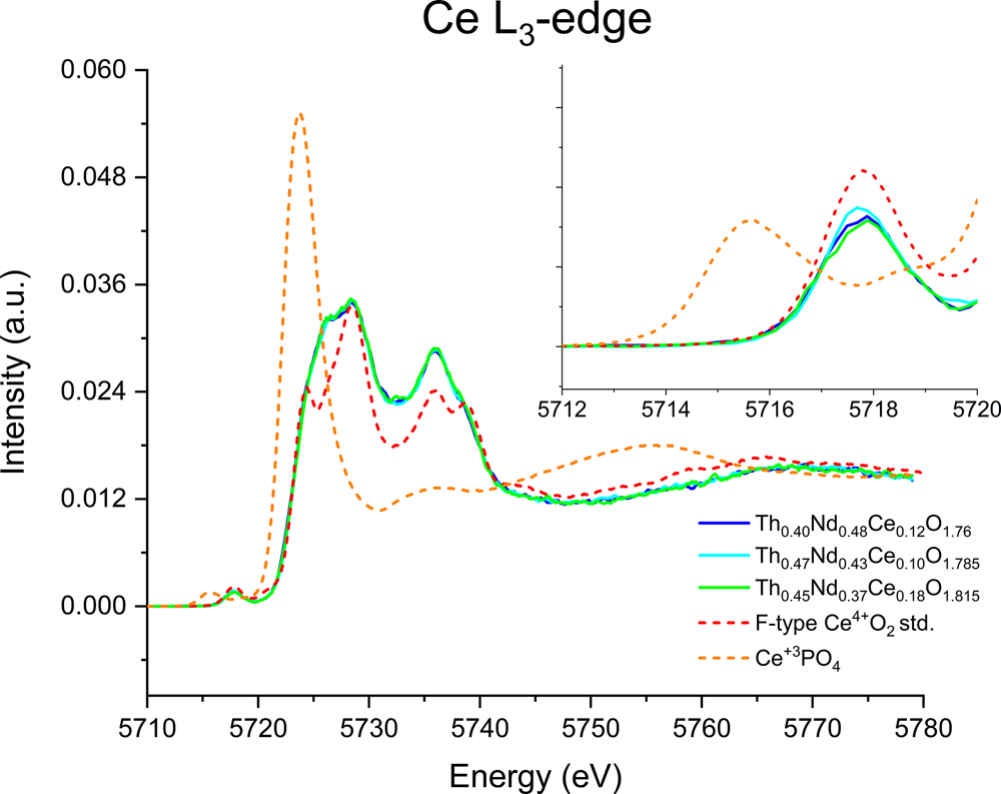
Normalized Ce L_3_-edge HERFD-XANES spectra in the range 5710 and 5780 eV for Th_0.40_Nd_0.48_Ce_0.12_O_1.76_, Th_0.47_Nd_0.43_Ce_0.10_O_1.785_, and Th_0.45_Nd_0.37_Ce_0.18_O_1.815_ with the standards F-type Ce^4+^O_2_ and Ce^3+^PO_4_. The inset details the pre-edge region for the spectra between 5712 and 5720eV.

It was identified from the S-PXRD analysis that the investigated compounds all exhibit characteristic superlattice reflections, which for the determined C-type bixbyite structure, is consistent with ordered oxygen vacancies.^16^ Although the samples were prepared under oxidizing conditions, it is not clear from diffraction analysis alone how charge balancing for the oxygen defects is achieved, via Nd^3+^ or whether reduced cerium states (Ce^4+^/Ce^3+^) are also present enabling this, of which is known to occur in other actinide bixbyite compounds.^16^ Additionally, other related oxide systems have been determined to possess reduced cationic states coupled with the occurrence of oxygen defect vacancies despite oxidizing conditions present during synthesis.^18−20^ Furthermore, the previous investigation by Nandi et al.^30^ did not perform XAS or similar analysis to conclusively determine the redox chemistry of Ce in the similar C-type bixbyite compounds they investigated. Consequently, HERFD-XANES measurements were first performed on the Ce L_3_-edge for Th_0.40_Nd_0.48_Ce_0.12_O_1.76_, Th_0.47_Nd_0.43_Ce_0.10_O_1.785_, and Th_0.45_Nd_0.37_Ce_0.18_O_1.815_ with Ce^3+^PO_4_ and Ce^4+^O_2_ standards to determine the Ce redox states present. Figure 3 depicts the normalized HERFD-XANES spectra on the Ce L_3_-edge from these measurements.

From Figure 3, the main edges for all three measured compositions, Th_0.40_Nd_0.48_Ce_0.12_O_1.76_, Th_0.47_Nd_0.43_Ce_0.10_O_1.785_, and Th_0.45_Nd_0.37_Ce_0.18_O_1.815_, were found to be consistent with the Ce^4+^O_2_ standard and not the Ce^3+^PO_4_ standard. This redox assignment is further supported in the pre-edge region, depicted in the inset of Figure 3, where the pre-edge peak at approximately 5717.5 eV corresponding to the 2p to mixed 5d–4f electron transition, which is characteristic of the tetravalent Ce^4+^ oxidation state, can be readily observed. Such a pre-edge peak for Ce^3+^ is known to occur at a lower energy, 5715.5 eV, as observed in the Ce^3+^PO_4_ standard displayed in Figure 3, and can be clearly seen to not match the title compounds, supporting their assignment of containing Ce^4+^.^41,42^ This demonstrates the presence of tetravalent cerium in all title compositions, and there is no evidence of trivalent cerium.^43^ Notably, the main-edge features differ considerably between the compounds and the F-type Ce^4+^O_2_ standard, and particularly sharp features in the standards spectra are absent in the studied compounds. Such blurring of the main-edge features for Ce has been previously observed in related F-type CeO_2_ where it was attributed to distortion and formation of clusters, leading to loss of near-neighbor Ce cations.^44^ This behavior of Ce electronic states and their effect on pre- and main-edge HERFD-XANES regions are well-documented and discussed in depth elsewhere.^44−46^

**Figure 4:**
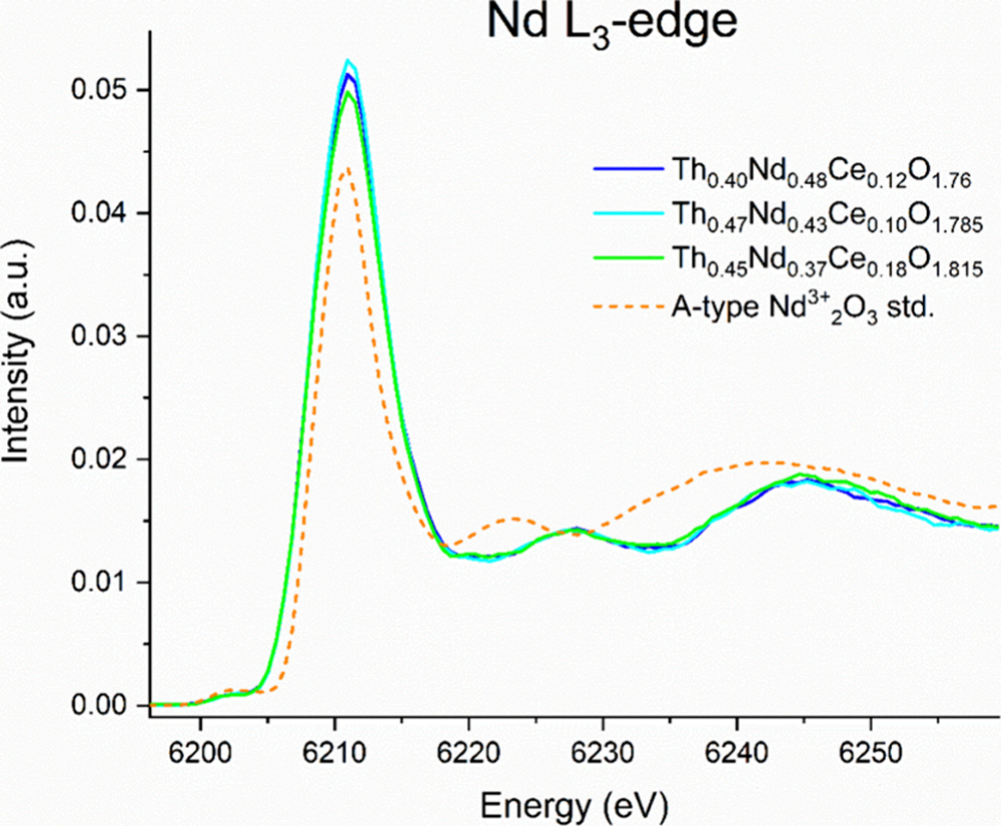
Normalized Nd L_3_-edge HERFD-XANES pre-edge region between 6196 and 6269 eV for Th_0.40_Nd_0.48_Ce_0.12_O_1.76_, Th_0.47_Nd_0.43_Ce_0.10_O_1.785_, and Th_0.45_Nd_0.37_Ce_0.18_O_1.815_ and with the standard A-type Nd^3+^_2_O_3_.

The Ce oxidation state in the Th_0.40_Nd_0.48_Ce_0.12_O_1.76_, Th_0.47_Nd_0.43_Ce_0.10_O_1.785_, and Th_0.45_Nd_0.37_Ce_0.18_O_1.815_ C-type bixbyite compounds is found to be consistently tetravalent, which implies that charge compensation for the oxygen vacancies must be achieved via the Nd^3+^ cations. To both unambiguously demonstrate this and further probe the local environment of Nd in Th_0.40_Nd_0.48_Ce_0.12_O_1.76_, Th_0.47_Nd_0.43_Ce_0.10_O_1.785_, and Th_0.45_Nd_0.37_Ce_0.18_O_1.815_, HERFD-XANES measurements were performed on the Nd L_3_-edge and are presented in Figure 4.

**Figure 5:**
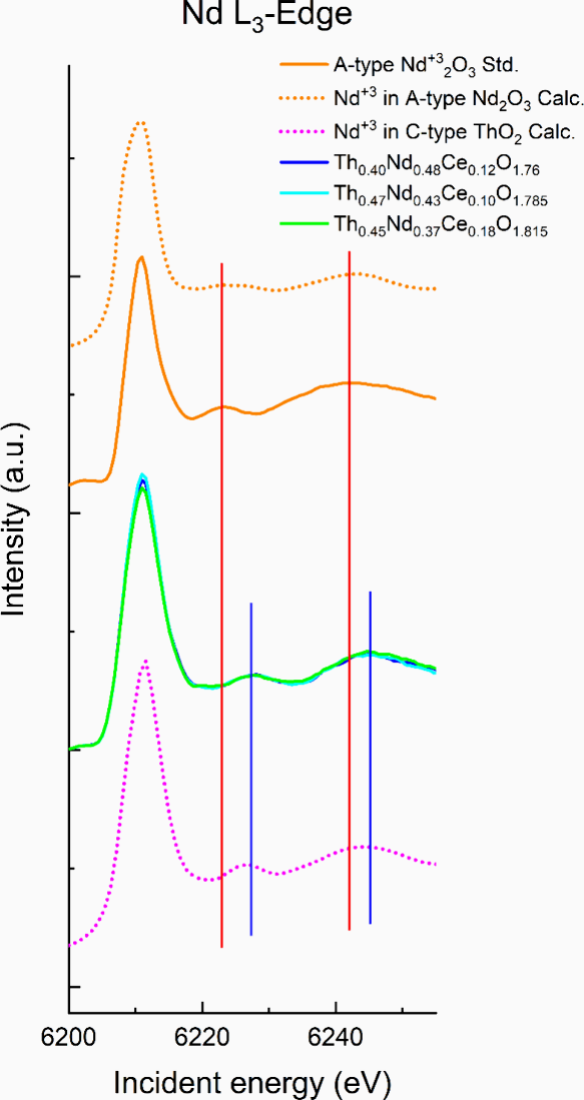
Nd L_3_-edge HERFD-XANES spectra for Th_0.40_Nd_0.48_Ce_0.12_O_1.76_, Th_0.47_Nd_0.43_Ce_0.10_O_1.785_, and Th_0.45_Nd_0.37_Ce_0.18_O_1.815_, standard Nd^3+^_2_O_3_ and simulated Nd^3+^ in C-type bixbyite ThO_2_ and Nd^3+^ in A-type Nd_2_O_3_ spectra using the FEFF9.6^38^ code. The red and blue vertical lines are guides to the differences in the post-edge regions. Highlighting the matching post-edge features between the measured samples and the theoretical Nd^3+^ in C-type bixbyite ThO_2_.

From Figure 4, the main Nd L_3_-edge for Th_0.40_Nd_0.48_Ce_0.12_O_1.76_, Th_0.47_Nd_0.43_Ce_0.10_O_1.785_, and Th_0.45_Nd_0.37_Ce_0.18_O_1.815_ follows closely that of the A-type Nd^3+^_2_O_3_ standard, indicating the as-expected presence of Nd^3+^ in these. However, inspection of the post-edge region shows considerable differences between the samples and the A-type Nd^3+^_2_O_3_ standard, suggesting the local environment differences around the Nd cations between samples and the standard. To understand this difference in local coordination behavior around the Nd^3+^ cations in Th_0.40_Nd_0.48_Ce_0.12_O_1.76_, Th_0.47_Nd_0.43_Ce_0.10_O_1.785_, and Th_0.45_Nd_0.37_Ce_0.18_O_1.815_, theoretical calculations were performed using FEFF9.6^38^ in which the Nd^3+^ cation was placed in a theoretical cubic structure of either C-type ThO_2_ or F-type CeO_2_ and also comparative trigonal sesquioxide A-type Nd_2_O_3_, and the HERFD-XANES spectra on the L_3_-edge for Nd were simulated, as shown in Figure 5.

From Figure 5 of the HERFD-XANES Nd L_3_-edge, the main and the postedge features of Th_0.40_Nd_0.48_Ce_0.12_O_1.76_, Th_0.47_Nd_0.43_Ce_0.10_O_1.785_, and Th_0.45_Nd_0.37_Ce_0.18_O_1.815_ are observed to align well with the simulated Nd^3+^ in C-type bixbyite ThO_2_ spectra, whereas they do not align with the standard A-type Nd^3+^_2_O_3_ and simulated Nd^3+^ in A-type Nd^3+^_2_O_3_. This indicates that the local environment of the Nd^3+^ cation in the Th_0.40_Nd_0.48_Ce_0.12_O_1.76_, Th_0.47_Nd_0.43_Ce_0.10_O_1.785_, and Th_0.45_Nd_0.37_Ce_0.18_O_1.815_ C-type bixbyite structures for their Nd^3+^ cations closely resembles that of C-type ThO_2_ compared to the more typical Nd_2_O_3_ A-type trigonal sesquioxide. In comparison to Ce^4+^ in Th_0.40_Nd_0.48_Ce_0.12_O_1.76_, Th_0.47_Nd_0.43_Ce_0.10_O_1.785_, and Th_0.45_Nd_0.37_Ce_0.18_O_1.815_, the Nd^3+^ cation, which provides charge compensation via oxygen defect formation, resides in the structure in a relatively relaxed and free from distortion manner.

### 3.3 Extended X-ray Absorption Fine Structure Spectroscopy

**Figure 6:**
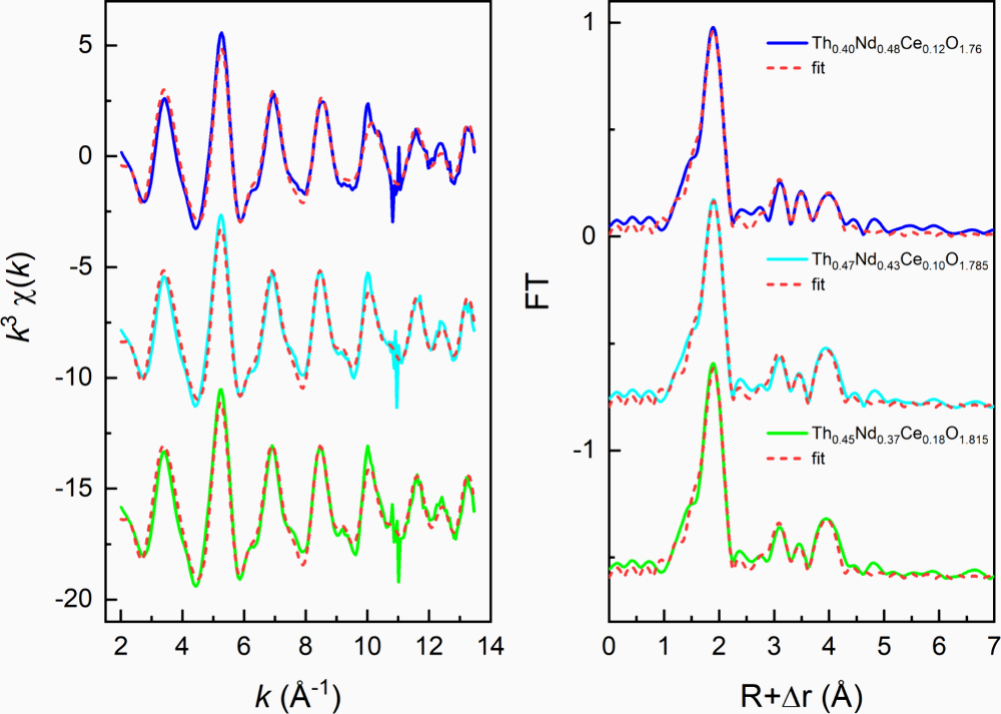
Fitted *k*^3^-weighted Th L_3_-edge EXAFS spectra (left) and corresponding Fourier transforms (FTs, right) for the C-type bixbyite compounds Th_0.40_Nd_0.48_Ce_0.12_O_1.76_, Th_0.47_Nd_0.43_Ce_0.10_O_1.785_, and Th_0.45_Nd_0.37_Ce_0.18_O_1.815._.

It has been demonstrated thus far that the compounds Th_0.40_Nd_0.48_Ce_0.12_O_1.76_, Th_0.47_Nd_0.43_Ce_0.10_O_1.785_, and Th_0.45_Nd_0.37_Ce_0.18_O_1.815_ adopt C-type bixbyite structures described in space group *Ia*3̅, in which oxygen vacancy defects are observed from S-PXRD measurements that are charged balanced via Nd^3+^, whereas Ce^4+^ occurs consistently. With respect to the literature,^30^ the formation of these phases can only be achieved when the cations are present as a ternary system, in which ternary formation does not appear to yield the C-type structure. HERFD-XANES measurements indicated that the local chemical environments of the Nd^3+^ and Ce^4+^ cations in the title compounds are relatively dissimilar, indicating variability in local order between them. Missing so far from this investigation is the role of the Th chemistry. Th^4+^ is not expected to possess any redox activity, and long-range should be randomly found across the cationic lattice of the bixbyite structure in the case of the ideal C-type bixbyite structure. The previous HERFD-XANES measurements described and emphasized the significance of the local structure and environment on the solid-state chemistry of the C-type bixbyite structures. The occurrence of ordered oxygen vacancy defects in the structure locally would likely be situated away from Th^4+^ to reduce unfavorable local reduction in the Th valence. To probe and examine this, EXAFS measurements were performed on the Th L_3_-edge. The fitted *k*^3^–weighted Th L_3_-edge EXAFS spectra and corresponding Fourier transforms (FTs) in the spectral range from 2.0 to 13.5 Å^–1^ are presented in Figure 6, and the results of the shell fit analysis are given in Table 1.

**Table 1:** EXAFS Fit Parameters for Th L_3_-Edge Data Measured from Th_0.40_Nd_0.48_Ce_0.12_O_1.76_, Th_0.47_Nd_0.43_Ce_0.10_O_1.785_, and Th_0.45_Nd_0.37_Ce_0.18_O_1.815_ in Addition to Th L_3_-edge XANES Data from Rothe et al.^47^ for ThO_2_^a^

sample	shell	CN	*R* (Å)	σ^2^ (Å)	ref
Th_0.40_Nd_0.48_Ce_0.12_O_1.76_	Th–O	6.0(2)	2.369(2)	0.0065(3)	present investigation
	Th–Th	5.0*	3.904(5)	0.0069(5)	present investigation
	Th–Nd	5.0*	3.847(6)	0.0081(7)	present investigation
	Th–Ce	2.0*	3.71(2)	0.013(3)	present investigation
	MS Th–O–Th–O′	6.0/—	4.738/—	0.0130/—	present investigation
	MS Th–O–O″	18.0/—	3.350/—	0.0130/—	present investigation
Th_0.47_Nd_0.43_Ce_0.10_O_1.785_	Th–O	5.9(2)	2.374(2)	0.0062(3)	present investigation
	Th–Th	6.0*	3.917(4)	0.0071(4)	present investigation
	Th–Nd	5.0*	3.849(7)	0.0089(8)	present investigation
	Th–Ce	1.0*	3.69(3)	0.011(3)	present investigation
	MS Th–O–Th-O′	5.9/—	4.748/—	0.0124/—	present investigation
	MS Th–O–O″	17.7/—	3.357/—	0.0124/—	present investigation
Th_0.45_Nd_0.37_Ce_0.18_O_1.815_	Th–O	6.1(2)	2.373(2)	0.0064(3)	present investigation
	Th–Th	6.0*	3.914(4)	0.0067(5)	present investigation
	Th–Nd	4.0*	3.860(7)	0.0075(1)	present investigation
	Th–Ce	2.0*	3.75(2)	0.013(4)	present investigation
	MS Th–O–Th–O′	6.1/—	4.746/—	0.0128/—	present investigation
		18.3/—	3.356/—	0.0128/—	present investigation
ThO_2_	Th–O	8.0	2.41	0.0054	Rothe et al.^47^
	Th–Th	12	3.98	0.0042	Rothe et al.^47^
	Th–O–O	24	4.63	0.0064	Rothe et al.^47^

a CN = coordination number, *R* = radial distance, σ^2^ = Debye–Waller factor, * = fixed parameter, /— linked parameter. Standard deviations of the variable parameters, as estimated by EXAFSPAK, are given in parentheses. The conservative common absolute error in the EXAFS shell fit is CN ± 20% and *R* ± 0.02 Å.^48^ Structural parameters of 2-fold degenerated 4- and 3-legged multiple scattering (MS) paths Th–O–Th–O′ and Th–O–O″ were linked to the fitted parameters of the first shell oxygen, respectively. Coordination numbers with an asterisk were fixed during fitting. *S*_0_^2^ was fixed to 0.9.

From the EXAFS analysis of the Th_0.40_Nd_0.48_Ce_0.12_O_1.76_, Th_0.47_Nd_0.43_Ce_0.10_O_1.785_, and Th_0.45_Nd_0.37_Ce_0.18_O_1.815_ compounds (Table 1), the Th–O bond distances were determined to range from 2.369 to 2.373 Å. These distances are shorter than the standard 2.41 Å previously found for ThO_2_, even when considering the absolute error of 0.02 Å for interatomic distances derived from EXAFS fitting.^47,48^ Similarly, in considering the specific EXAFS measurements of the Th_0.40_Nd_0.48_Ce_0.12_O_1.76_, Th_0.47_Nd_0.43_Ce_0.10_O_1.785_, and Th_0.45_Nd_0.37_Ce_0.18_O_1.815_ compounds, the Th–Th distance of 3.904–3.917 Å was also found to be contracted compared to 3.98 Å^47^ in ThO_2_.^49^ The contraction is observed to be most pronounced for Th_0.40_Nd_0.48_Ce_0.12_O_1.76_, which contains the greatest amount of Nd. Interestingly, the same sample also presents the largest lattice expansion from the S-PXRD analysis (Figure 2). This variable chemical behavior is understood to arise from the Nd^3+^ cation which first has a larger ionic radius than Th^4+^ (1.109 vs 1.05 Å) for CN = 8, leading to lattice expansion.^50^ Concurrently, the Nd^3+^ cation, as shown through HERFD-XANES analysis, also induces the formation of oxygen vacancy defects. It has been previously established^51,52^ that the occurrence of oxygen vacancies in a crystal lattice due to the inclusion of a trivalent cation within a tetravalent cationic lattice network results in electrostatic attraction of anions toward the oxygen vacancy positions. This leads to local bond contraction around the vacancy via anions, despite a global expansion of the lattice network for similar cations. The observations from S-PXRD and EXAFS measurements for the C-type bixbyite structures Th_0.40_Nd_0.48_Ce_0.12_O_1.76_, Th_0.47_Nd_0.43_Ce_0.10_O_1.785_, and Th_0.45_Nd_0.37_Ce_0.18_O_1.815_ are consistent with these previous observations and measurements.^51,52^

To draw further conclusions on the local coordination environment of the dopant cations, a hypothetical Th–O and Th–M (M = Th, Nd, Ce) distance based on a linear combination of crystallographic M–O or M–M distances in the parent oxide structures, namely, F-type ThO_2_, F-type CeO_2_, and A- or C-type Nd_2_O_3_, can be calculated. In other words, this approach uses the targeted stoichiometry of the C-type bixbyite solids, to derive an average Th–Th and Th–M distance that is based on the ideal crystal structures of the individual components in the C-type bixbyite solid solution. When comparing these calculated distances with the experimental ones, indications for how similar or dissimilar the solid solution environments are from the end-member oxide structures. The crystallographic data for the reference compounds (end-member oxides) are summarized in Table 2.

**Table 2:** Average Crystallographic M–O and M–M Distances in ThO_2_, CeO_2_, and Nd_2_O_3_

crystal structure	M–O_av_ (Å)	M–M_av_ (Å)	ref
ThO_2_	2.425	3.960	Idiri et al.^53^
CeO_2_	2.370	3.871	Hull et al.^54^
A-type Nd_2_O_3_	2.480	3.775	Faucher et al.^55^
C-type Nd_2_O_3_	2.375	3.694	Bommer^56^

The A-type Nd_2_O_3_ phase has a M–O bond clearly longer than that of any of the other oxide structures. Based on obtained bond lengths from our EXAFS data, the sample with the highest Nd content, i.e., Th_0.40_Nd_0.48_Ce_0.12_O_1.76_ yields an average Th–O bond of 2.369 Å. Calculating a Th–O distance based on the crystallographic bond lengths in the individual oxides would yield a bond length of 2.394 Å, assuming C-type Nd_2_O_3_ and 2.445 Å for A-type Nd_2_O_3_. The former bond length is closer to the measured one, implying that the local Nd^3+^ environment is closer to C-type Nd_2_O_3_ or F-type CeO_2_ than to A-type Nd_2_O_3_, in good agreement with the HERFD-XANES results discussed previously. The additional contraction of the measured Th–O bond in the bixbyite phases is likely due to the presence of oxygen vacancies in the lattice. Finally, the measured Th–O distance of 2.373 Å in the sample with the highest Ce-content, i.e., in Th_0.45_Nd_0.37_Ce_0.18_O_1.815_ is very similar to the crystallographic Ce–O bond length in CeO_2_. However, the Th–Ce distance of 3.75 Å in this sample is significantly shorter than the Ce–Ce distance in CeO_2_, which would imply that the Ce^4+^ environment in the bixbyite structure is rather different from the Ce coordination environment in the F-type CeO_2_ structure, consistent with the HERFD-XANES measurements.

It is somewhat surprising that the local environment around the Th^4+^ cation is different to what is found in regular F-type ThO_2_, in which the observed bond contraction induced via oxygen vacancy defects will enhance the cation valence. The calculation of the Th bond valence sums (BVS) in the investigated compounds highlights this, where using the Th–O bond lengths from the EXAFS analysis for Th_0.40_Nd_0.48_Ce_0.12_O_1.76_, Th_0.47_Nd_0.43_Ce_0.10_O_1.785_, and Th_0.45_Nd_0.37_Ce_0.18_O_1.815_ with the values for Brese and O’Keeffe^57^ returns respective values of 4.60, 4.55, and 4.55 compared to 4.15 for ThO_2_. This further emphasizes the considerably different and significant local chemical environments of not just Th but also Ce and Nd found within the C-type bixbyite structures of Th_0.40_Nd_0.48_Ce_0.12_O_1.76_, Th_0.47_Nd_0.43_Ce_0.10_O_1.785_, and Th_0.45_Nd_0.37_Ce_0.18_O_1.81_. The formation of these phases appears to be most dependent upon the occurrence of oxygen vacancy defects and subsequently, as measurements demonstrate, due to the occurrence of Nd^3+^. The Ce cation appears to play a relatively insignificant role in terms of redox, not inducing defect formation due to occurring only as Ce^4+^ and only contributing to subtle lattice contraction, having a smaller ionic radii than Th^4+^.^50^ However, as shown from the HERFD-XANES measurements on the Ce L_3_-edge, the Ce^4+^ cation exists in the Th_0.40_Nd_0.48_Ce_0.12_O_1.76_, Th_0.47_Nd_0.43_Ce_0.10_O_1.785_, and Th_0.45_Nd_0.37_Ce_0.18_O_1.815_ structures with a heightened degree of distortion and apparent local clustering formation.^44^ Nevertheless, the interplay of Th^4+^, Nd^3+^, and Ce^4+^ is still significant, since the ternary configurations of these, Th_1–*x*_Nd_*x*_O_2–0.5_*x*, Th_1–*x*_Ce_*x*_O_2–0.5*x*_, and Ce_1–*x*_Nd_*x*_O_2–0.5*x*_^30^, with respect to the literature do not appear to yield the C-type bixbyite structure. Consequently, it is only when encountered as a ternary cationic system, Th_1–*x_–_y*_Nd_*x*_Ce_*y*_O_2–0.5*x*_, that the C-type bixbyite structure be obtained and emerges.^30^ This suggests the significance of configurational entropy in cation mixing between constituents in assisting in structure stabilization of the C-type bixbyite, often described in related high entropy oxide (HEO) materials.^58^ Although the investigated C-type bixbyite structures are perhaps too simple to be considered in the HEO class with respect to their number of cation components, the heightened complexity found in SNF suggests that similar structures may be found and stabilized through cation mixing.

## 4 Conclusions

The C-type bixbyite structures Th_0.40_Nd_0.48_Ce_0.12_O_1.76_, Th_0.47_Nd_0.43_Ce_0.10_O_1.785_, and Th_0.45_Nd_0.37_Ce_0.18_O_1.815_ have been shown using Le Bail refinements against S-PXRD measurements to adopt C-type bixbyite structures in space group *Ia*3̅, possessing characteristic superlattice reflections consistent with oxygen vacancy defects. HERFD-XANES measurements on the Ce and Nd L_3_-edge demonstrated that charge balancing is achieved through Nd^3+^ where possible reduced cerium is precluded, with only Ce^4+^ measured in the compositions. Careful inspection and analysis of the HERFD-XANES spectra supported by electronic structure calculations for the compounds on the Ce and Nd L_3_-edge Ce and Nd L_3_-edges revealed subtly different but significantly contrasting local environments for these cations within the Th_0.40_Nd_0.48_Ce_0.12_O_1.76_, Th_0.47_Nd_0.43_Ce_0.10_O_1.785_, and Th_0.45_Nd_0.37_Ce_0.18_O_1.815_ C-type bixbyite structures. EXAFS measurements highlight the effect of local bond contraction induced via oxygen defect formation through Nd^3+^ inclusion, resulting in enhanced Th bond valence despite bulk lattice expansion. These observations were further supported by linear combination calculations of the M–O bond lengths of the Th–O bond lengths in studied compounds and reference ThO_2_/C-type/A-type Nd_2_O_3_/F-typeCeO_2_ which further demonstrate the role of oxygen defects via Nd^3+^ incorporation, leading to local bond contraction. The significance of cation mixing and configurational entropy has been further highlighted, whereby the investigated C-type bixbyite structures are not known to form as ternary systems but are only obtained when synthesized as quaternary. Often thought to be driven by the interplay of oxygen defects,^16^ this investigation highlights the pertinence of local cation chemistry in influencing the phase transformation from F-type to C-type in doped actinide oxides. Finally, the study points toward the general significance of variability in short-range order and chemistry that arises from dissimilar cations which are mixed within long-range disordered oxide structures, of those particularly relevant to nuclear waste management.
